# Effects of Respiratory Disorders on Smoking Cessation and Re-Initiation in an Italian Cohort Study

**DOI:** 10.3390/ijerph18030903

**Published:** 2021-01-21

**Authors:** Eliana Finocchio, Mario Olivieri, Giang Nguyen, Oscar Bortolami, Pierpaolo Marchetti, Roberta Vesentini, Lorena Torroni, Gianluca Spiteri, Francesca Locatelli, Francesca Moretti, Alessandro Fois, Pietro Pirina, Marcello Ferrari, Giuseppe Verlato

**Affiliations:** 1 Unit of Epidemiology & Medical Statistics, Department of Diagnostics & Public Health, University of Verona, 37134 Verona, Italy; eliana.finocchio@univr.it (E.F.); giang.nguyen@univr.it (G.N.); oscar.bortolami@univr.it (O.B.); pierpaolo.marchetti@univr.it (P.M.); roberta.vesentini@univr.it (R.V.); lorena.torroni@univr.it (L.T.); francesca.locatelli@univr.it (F.L.); giuseppe.verlato@univr.it (G.V.); 2 Unit of Occupational Medicine, Department of Diagnostics & Public Health, University of Verona, 37134 Verona, Italy; gianluca.spiteri@univr.it; 3 Unit of Hygiene, Department of Diagnostics & Public Health, University of Verona, 37134 Verona, Italy; francesca.moretti@univr.it; 4 Unit of Lung Disease, Department of Clinical and Experimental Medicine, University of Sassari, 07100 Sassari, Italy; agfois@uniss.it (A.F.); pirina@uniss.it (P.P.); 5 Unit of Respiratory Diseases, Department of Medicine, University of Verona, 37134 Verona, Italy; marcello.ferrari@univr.it

**Keywords:** smoking cessation, smoking re-initiation, asthma, allergic rhinitis, chronic bronchitis, smoking intensity, abstinence duration

## Abstract

The present study aims to prospectively assess the influence of respiratory disorders on smoking cessation and re-initiation. Three population-based Italian cohorts answered a questionnaire on respiratory health and smoking habits during 1998–2001 and after a mean follow-up (SD) of 9.1 (0.8) years. Out of 1874 current smokers and 1166 ex-smokers at baseline, 965 (51.5%) and 735 (63.0%) reported their smoking status at follow-up. From current smokers, 312 had stopped smoking at follow-up, while 86 ex-smokers had resumed smoking. People reporting asthma at baseline were more likely to stop smoking than the other subjects (48.6% vs. 31.7%), while people reporting allergic rhinitis or chronic cough/phlegm had a higher probability to resume smoking (16.7% vs. 10.5% and 20.7% vs. 10.4%, respectively). In the multivariable logistic model, smoking relapse strongly decreased with increasing abstinence duration in people without chronic cough/phlegm (OR for ≥7.5 years vs. <7.5 years = 0.23, 95% CI 0.20–0.27), while no effect was detected in people with chronic cough/phlegm (*p* for interaction = 0.039). Smoking cessation was enhanced in asthmatic subjects, while people with allergic rhinitis or chronic cough/phlegm were at higher risk to resume smoking. Chronic cough/phlegm blunted the decrease in smoking resumption associated with longer abstinence duration.

## 1. Introduction

The prevalence of tobacco smoking has been decreasing in most of the world for the last decade, as the result of the balance between smoking cessation and smoking initiation rates indicates [1,2,3]. In order to implement effective anti-smoking policies among adults, it is necessary to analyse the determinants of smoking cessation and re-initiation in the population thoroughly. Smoking cessation is a complex phenomenon involving psychological [4], social [5,6], and healthcare factors [7]. The role of health status is still a matter of debate. Some studies reported that health concerns are the main motivation to stop smoking [8], including concern for foetal health in pregnant women [9]. However, the influence of respiratory disorders on smoking cessation is still controversial. Smoking prevalence almost halved among people with asthma during a seven-year period in a Swedish cohort study [10]. On the other hand, diagnosis of respiratory disease did motivate people to quit smoking in the longitudinal Respiratory Health in Northern Europe study (RHINE) [11]. Likewise, smokers with asthma, attending the North American Quitline Consortium (NAQC), were less likely to quit than the other smokers [12]. As regards COPD, smoking cessation has been reported to be more difficult among smokers with than without the disease [13]. Moreover, chronic respiratory diseases, such as asthma and COPD, are associated with an increase in quit attempts [12,14], and in the willingness to stop smoking [15,16]. Remembering Mark Twain’s joke—‘It’s easy to quit smoking. I’ve done it a thousand times’—these findings seem to suggest that chronic respiratory diseases could increase the risk of smoking relapse. To our knowledge, the relation between smoking re-initiation and respiratory diseases has not been addressed yet.

To properly address the relation between chronic respiratory disorders and smoking cessation and re-initiation, it should be reminded that these processes are affected by several other factors. The probability to stop smoking has been reported to be inversely related to the number of cigarettes smoked daily [12], age at smoking initiation [17], and smoking duration [17,18]. Smoking re-initiation increased with decreasing age at smoking cessation [19], duration of smoking abstinence [19,20,21], and actual age [19,21,22,23]. In particular, the youngest individuals resumed smoking earlier than older adults, mainly under the influence of friends [24]. In addition, lower education level [23], affective, emotional, and situational processes [22,25], as well as smoking behaviour in social networks [26,27], directly influenced the relapse. In most cases, the success of smoking cessation strategies was not followed by effective relapse prevention [28], so further studies are needed to identify factors affecting smoking cessation [29] and re-initiation. The aim of the present study was to evaluate the influence of respiratory disorders on smoking cessation and re-initiation, taking into account also baseline smoking characteristics.

## 2. Materials and Methods

### 2.1. Study Design

Smoking cessation and re-initiation, and their related determinants, were investigated in three population-based cohorts recruited in Italy between 1998 and 2001. The enrolment and follow-up of the three cohorts have been previously described [30]. Briefly, two cohorts had been enrolled in the frame of the Italian Study on Asthma in Young Adults (ISAYA) in Verona (Northern Italy) and Sassari (Mediterranean island of Sardinia) [31], while the third cohort had been recruited in Verona in the frame of the Italian Study on the Incidence of Asthma (ISIA) [32]. At the baseline, the three cohorts comprised 5933 subjects overall, who had answered a screening questionnaire on socio-demographic characteristics, respiratory disorders, smoking habits, and socio-economic burden [33]. The Verona and Sassari-ISAYA cohorts comprised respectively 2166 and 2055 subjects aged 20–46 years, while the Verona-ISIA cohort was slightly older, comprising 1712 subjects aged 28–54 years. The three cohorts were contacted again during 2007–2009, after a follow-up of 9.1 ± 0.8 years (mean ± SD), in the frame of the Gene-Environment Interactions in Respiratory Diseases (GEIRD) [34], whose protocol is available at www.geird.org. Subjects were administered the GEIRD screening questionnaire [34] by mail, containing the same questions on respiratory disorders and smoking habits used at the baseline. Non-responders were contacted again, first by mail and then by phone.

### 2.2. Assessment of Smoking Habits

Subjects were classified as (1) current smokers if they reported having smoked at least one cigarette per day or one cigar a week for as long as one year and also in the last month; (2) ex-smokers if they had smoked the same minimum amount previously reported but had stopped smoking for at least one month before the interview; and (3) never smokers otherwise. To investigate smoking cessation and its determinants, the study focused on current smokers at the baseline, who were re-evaluated at the follow-up to determine whether they were persistent smokers or had quit smoking for at least one month before the interview. To investigate smoking re-initiation and its determinants, the study focused on ex-smokers at the baseline, who were re-evaluated at the follow-up to determine whether they were persistently abstinent from smoke or had relapse smoking. Baseline smoking intensity was assessed as the number of cigarettes smoked daily. Cumulative smoke exposure in pack-years was computed as years of smoking (the difference between the age at interview and the age when smoking started) multiplied by the average daily consumption of 20-cigarette packs. Two additional variables—the age at smoking initiation and years of smoking abstinence until the baseline—were analysed, respectively, as determinants of smoking cessation and re-initiation. Self-reported smoking status had been validated in the European Community Respiratory Health Survey (ECRHS) performed in Verona during 1991–1993, where a good agreement (Cohen’s κ = 0.93) had been found between self-reported smoking and serum cotinine levels [35].

### 2.3. Assessment of Respiratory Disorders

Current asthma was assumed when a subject affirmatively answered the question ‘Have you had one asthma attack in the last 12 months” and/or “Are you currently taking any medicine for asthma?’ Allergic rhinitis was defined by a positive answer to the question, ‘Do you have any nasal allergies including hay fever?’ and chronic cough/phlegm by a positive answer to the question, ‘Have you had cough and phlegm on most days for a minimum of three months a year and for at least two successive years?’

### 2.4. Statistical Analyses

Significances of differences in response to follow-up questionnaire or smoking cessation/re-initiation were evaluated by Fisher’s exact test or chi-squared test for nominal variables, chi-square for trend for ordinal variables, and Wilcoxon–Mann–Whitney test for skewed distributed continuous variables. The 95% confidence interval of cumulative incidence was computed by the binomial exact method. Potential determinants of smoking cessation and re-initiation were further evaluated in multivariable analysis, which was accomplished by logistic regression models, where smoking cessation or re-initiation were the response variables. When addressing smoking cessation, centre, gender, age class (20–31.2 years old, 31.3–42.5 years old, and 42.6–53.9 years old), asthma (or allergic rhinitis or chronic cough/phlegm), cigarettes smoked daily (1–5, 6–10, 11–15, and 16–40) and age at smoking initiation (8–15 years old, 16–17 years old, 18–19 years old, and > = 20 years old) were the explanatory variables. When considering smoking re-initiation, age at the time of smoking initiation was replaced by years of smoking abstinence until the baseline (<3 years, 3–7.4 years, 7.5–13.4 years, and >13.5 years) in the logistic model. Respiratory disorders were separately introduced into different models because they were strongly collinear [36]. The interaction between respiratory disorders and smoke-related variables was also tested. Standard errors were adjusted for intra-cohort correlation. The goodness of fit of logistic models was tested by the Hosmer–Lemeshow test [37] and the significance of model parameters was tested by the Wald test. The analyses were performed in Stata 14 (StataCorp, College Station, TX, USA) and statistical significance was set at *p* < 0.05.

## 3. Results

### 3.1. Response to Follow-Up

Of the initial cohort of 5933 subjects, 3597 (60.6%) answered the follow-up questionnaire during 2007–2009 after a mean (SD) follow-up of 9.1 (0.8) years. The response was significantly affected by the type of cohort and smoking habits. A different mix of responses was observed among the three cohorts: Verona cohorts achieved a higher response percentage (1325/1712 = 77.4% and 1459/2166 = 67.4%, respectively, for Verona-ISIA and Verona-ISAYA) than the Sassari cohort (813/2055 = 39.6%). Moreover, response percentage was the highest among ex-smokers (761/1166 = 65.3%), intermediate among never smokers (1808/2877 = 62.8%), and the lowest among current smokers (1027/1874 = 54.8%) (*p* < 0.001).

### 3.2. Changes in Smoking Habits

Smoking habits at follow-up were not reported by 114 subjects, namely, by 26 never smokers, 26 ex-smokers, and 62 current smokers. Incidence and determinants of smoking cessation and re-initiation were investigated in responders to the item on smoking habits at follow-up item, i.e., on 965 and 735 subjects, who were respectively current smokers and ex-smokers at baseline. During the follow-up, smoking cessation largely exceeded smoking initiation. Indeed, 312 subjects out of 965 current smokers at baseline (32.3%, 95% CI 29.4–35.4%) had stopped smoking at the follow-up examination, while 18 subjects out of 1782 never smokers (1.0%, 95% CI 0.6–1.6%) had started smoking, and 86 out of 735 ex-smokers (11.7%, 95% CI 9.5–14.2%) had resumed smoking. In addition, two subjects had started and stopped smoking during follow-up.

### 3.3. Possible Selection Bias in Participation to the Follow-Up

Selection bias, possibly arising from low response rate, was evaluated by comparing baseline characteristics of subjects participating and not participating in the follow-up. As shown in Table 1, response to follow-up was higher in the Verona cohorts and in women, and it increased with increasing age both in baseline current smokers and ex-smokers. As regards occupation, participation was the highest among clerks, workmen, and housewives, and the lowest among unemployed. Smokers with chronic cough/phlegm were less likely to participate in the follow-up, while nasal allergies and asthma did not influence participation. Compared to responders to follow-up, non-responders smoked more cigarettes per day at baseline among current smokers and had been abstaining from smoking for less period of time among ex-smokers.

### 3.4. Determinants of Smoking Cessation

Cumulative incidence of smoking cessation decreased with advancing age (Table 2). Accordingly, quitting smoking more frequently occurred in the younger ISAYA cohorts than in the older ISIA cohort. Smokers’ occupation markedly influenced smoking cessation, whose cumulative incidence ranged from 20–25% among housewives and retired/others to nearly 50% among students. People suffering from asthma were more likely to stop smoking, while allergic rhinitis and chronic cough/phlegm did not significantly affect smoking cessation. As regards baseline smoking habits, the cumulative incidence of smoking cessation significantly increased as the number of cigarettes smoked daily and the pack-years decreased.

**Figure 1:**
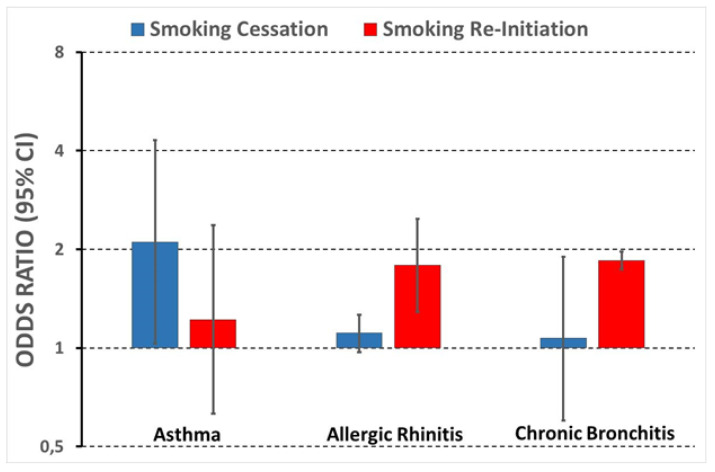
The Odds Ratio (OR) of smoking cessation and re-initiation as a function of respiratory disorders, investigated among current smokers and ex-smokers at baseline, respectively. ORs of smoking cessation and related *p-*values were obtained by a logistic model, controlling for centre, sex, age, number of cigarettes smoked daily, and age of smoking initiation. When investigating smoking re-initiation, age at smoking initiation was replaced in the logistic model by years of smoking abstinence until baseline.

Results of the univariable analysis were confirmed by multivariable analysis. The odds of smoking cessation significantly decreased with increasing smoking intensity (Table 3). When considering cumulative smoking exposure, the effect became even stronger—with respect to a cumulative smoking exposure <5 pack-years, the odds ratios (ORs) of smoking cessation significantly decreased to 0.74 (0.59–0.94) and 0.38 (0.25–0.58), respectively, in people who had smoked 10–14 pack-years and > = 15 pack-years. Smoking cessation was not significantly affected by age at smoking initiation. As regards respiratory disorders, asthma was associated with a two-fold increase in the probability of smoking cessation (Table 3 and Figure 1), while allergic rhinitis and chronic cough/phlegm had no effect, the ORs being 1.11 (0.97–1.26; *p* = 0.130) and 1.07 (0.60–1.90; *p* = 0.822), respectively. Of note, a significant interaction was detected between age at smoking initiation and chronic cough/phlegm (*p* = 0.024). The odds of smoking cessation decreased with increasing age of smoking initiation in subjects reporting chronic cough/phlegm (OR of ≥18 years versus <18 years = 0.61, 95% CI 0.30–1.23), while remaining about constant in the other subjects (OR = 1.09, 0.87–1.37). In addition, smoking cessation was significantly higher in the ISAYA-cohorts and lower in women and in people aged 31.3–42.6 years old.

### 3.5. Determinants of Smoking Re-Initiation

Cumulative incidence of smoking re-initiation decreased with advancing age, as it did for smoking cessation: the higher probability of smoking relapse was observed in the age class of 20–31.3 years (Table 4). People suffering from allergic rhinitis and chronic cough/phlegm were more likely to relapse smoking, while asthma did not significantly affect smoking re-initiation. As regards baseline smoking habits, the cumulative incidence of re-initiation exponentially increased with shortening smoking abstinence assessed at baseline, while age at smoking initiation, number of daily cigarettes, and pack-years smoked before abstinence did not affect smoking re-initiation.

Results of the univariable analysis were confirmed by multivariable analysis. The odds of smoking re-initiation were significantly lower in the Verona-ISAYA cohort and not significantly affected by sex (Table 5). People aged 42.6–53.9 years old were less likely to relapse during follow-up compared to people aged 20–31.2 years old. The risk of smoking relapse was inversely related to abstinence duration and tended to increase with the increase in the number of cigarettes smoked daily. As regards respiratory disorders, allergic rhinitis and chronic cough/phlegm significantly increased the likelihood of smoking re-initiation, the ORs being 1.79 (1.29–2.48; *p* < 0.001) and 1.85 (1.74–1.97; *p* < 0.001), respectively, whereas asthma had no effect (OR = 1.22, 0.63–2.37; *p* = 0.593) (Figure 1). Of note, an interaction between chronic cough/phlegm and abstinence duration was detected (*p* = 0.039). Among people not reporting chronic cough/phlegm, those who had abstained from smoking for ≥7.5 years had a four-fold lower risk to resume smoking than people with shorter abstinence duration (OR = 0.23, 0.20–0.27; *p* < 0.001), while no difference was detected among subjects with chronic cough/phlegm (OR = 0.92. 0.29–2.85; *p* = 0.880). The effect of abstinence duration on smoking resumption was not modified by allergic rhinitis (*p* = 0.870); indeed, the OR to resume smoking in people with ≥7.5 years of smoking abstinence versus <7.5 years was 0.28 (0.18–0.44) in people not reporting allergic rhinitis and 0.31 (0.15–0.63) in people reporting the disease.

Odds Ratios with 95% confidence intervals and *p-*values were computed by a multivariable logistic model. *P-*values were computed by the Wald test. Significant results are highlighted in bold.

## 4. Discussion

In the present longitudinal study, 312 out of 965 current smokers stopped smoking during a nine-year follow-up, and 86 out of 735 ex-smokers resumed smoking, yielding enough power to highlight the present findings:

1. The smoking intensity and cumulative smoke exposure were inversely related to smoking cessation, which represented its most important predictors in agreement with the current literature [12,17,18]. On the opposite side, the duration of smoking abstinence was inversely related to smoking re-initiation and was the most important predictor according to the current literature [19,20,21].
2. As regards respiratory disorders, the probability to quit smoking was affected neither by allergic rhinitis nor chronic cough/phlegm, while being doubled by current asthma. Conversely, the risk to re-start smoking increased almost twice in people with allergic rhinitis or chronic cough/phlegm, while being unaffected by asthma. Chronic cough/phlegm seemed to blunt the decrease in smoking resumption associated with longer abstinence duration.
3. People aged 20–31 years old had a higher probability to quit smoking than older people which, however, was partly counterbalanced by a much higher probability to resume smoking.

### 4.1. Determinants of Smoking Cessation

In an Italian national survey, concern for own health status was the most important reason to stop smoking, where 43.2% of ex-smokers had stopped smoking for a current health condition and an additional 31.9% to avoid future health problems [8]. At variance, the Italian behavioural risk factor surveillance system (PASSI) study reported that the presence of at least one chronic disease or a bad health status did increase quit attempts, but not successful ones [38], in agreement with American surveys [12,15,16]. The present study supports the role of health status in promoting smoking cessation. However, only a severe disease such as asthma increased the probability of quitting smoking, while milder diseases, such as rhinitis or chronic cough/phlegm, had no effect. Accordingly, in an observational Swedish study [10], smoking prevalence almost halved during a seven-year period in patients with asthma, while remaining unchanged in patients with COPD. Of note, while chronic cough/phlegm did not affect smoking cessation in the present study, other studies reported that COPD is even associated with a decrease in quitting smoking [12,13]. At variance, an American survey on Quitline users found that asthma does not increase but rather decreases smoking cessation [12]. Men were slightly more likely to stop smoking than women. According to previous longitudinal [39] or retrospective [38,40] studies, the present longitudinal study confirmed that older adults had a lower probability to stop smoking compared to younger adults.

### 4.2. Determinants of Smoking Re-Initiation

The present study found a significant effect of respiratory health status on smoking relapse—subjects with milder respiratory conditions, such as rhinitis or chronic cough/phlegm, had an increased risk of relapse with respect to controls, while current asthma had no effect. It could be hypothesised that people with serious respiratory diseases, such as current asthma, are more likely to quit smoking and less likely to relapse because they have more concerns for their health. On the other hand, subjects with mild respiratory symptoms could perform more unsuccessful attempts to quit. Indeed, chronic respiratory diseases are associated with an increase in quit attempts [12,14], which seem less likely to succeed [12,13]. To our knowledge, no literature data were available on the relation between smoking re-initiation and respiratory diseases.

Age was found to inversely influence smoking resumption [19,21,22,23,39]. In a Swiss prospective study, compared to the age class 35–44 years old, the ORs of smoking relapse progressively decreased to 0.60, 0.37, and 0.20, respectively, in people aged 45–54 years old, 55–64 years old, and 65–75 years old [39]. An American longitudinal study combining datasets from four population-based studies found that the risk of relapse was more than halved in people aged 25–64 years old with respect to youngsters aged 18–24 years old [22]. In the National Epidemiologic Survey of Alcohol and Related Conditions (NESARC) study, the younger age at cessation and shorter duration of abstinence variables predicted risk for relapse in the general population [19]. In a longitudinal survey of 1296 ex-smokers recruited as part of the International Tobacco Control (ITC) Four Country Survey (Australia, Canada, UK, and the USA), the likelihood to resume decreased for adults more than 40 years old compared to subjects aged 18–24 years old [21]. Our data confirmed age as having an important influence on smoking relapse because the highest and lowest percentages of smoking relapse were observed in the youngest (20–31 years old) and oldest (43–54 years old) age classes, respectively.

The present study found that smoking abstinence duration at baseline was inversely related to smoking re-initiation according to the current literature. Indeed, in the National Epidemiologic Survey of Alcohol and Related Conditions (NESARC) study, a shorter duration of abstinence predicted the risk for relapse in the general population [19]. In addition, according to the ITC survey, the proportion of relapse decreased from 78% in subjects with 1–7 days of abstinence to 5% in people with >730 days of abstinence [21]. Furthermore, in a community-based, longitudinal cohort, the proportion of sustained smoking abstinence increased with increasing duration of abstinence assessed at the baseline [20].

### 4.3. Strengths and Limitations

The present study has several strengths. First, although relapsing is very common after quitting, limited knowledge on smoking re-initiation is available for the general population, as many predictors of smoking relapse have been identified among treatment-seeking smokers in clinical samples [25,28]. The present study, instead, was conducted on three population-based cohorts recruited from the Italian general population. Second, most studies on smoking cessation were carried on adolescents or young adults [39], while studies on middle-aged people are sparse. Moreover, most studies performed in Italy were either cross-sectional [38] or retrospective [8], while the present study was a prospective cohort study. Furthermore, all studies on determinants of smoking re-initiation focused on psychological and social factors, alongside age and abstinence duration. To our knowledge, the present study is the first to investigate respiratory diseases as potential determinants of resume smoking.

The main limitation was the low response to follow-up among current smokers. Although non-responders were re-contacted twice by mail and by phone, only 54.8% of current smokers answered the follow-up questionnaire, and only 51.5% filled in questions on smoking habits. These percentages were higher for ex-smokers—65.3% of them answered the follow-up questionnaire and 63% filled in questions on smoking habits. However, no response, while largely affecting prevalence estimates [41], should not affect associations between determinants and outcome [42]. So, the estimate of cumulative incidence should be interpreted with caution, while the measures of association can be taken more confidently. Another limitation is the lack of information about e-cigarettes use, which seems to favour smoking relapse among former smokers [43]. However, it should be reminded that the use of e-cigarettes and other tobacco products was rare during 1998–2001, i.e., when baseline information was collected in the present cohort study.

## 5. Conclusions

Smoking cessation was enhanced in people with asthma, but not in people with allergic rhinitis or chronic cough/phlegm, who were even more likely to resume smoking after a successful attempt. Quitting smoking was more common among light smokers, while relapsing was more common among the youngest and being few years’ abstinent ex-smokers. So far, the success of smoking cessation strategies is not paralleled by efficient relapse prevention [28]. To be effective, public health policies aimed at reducing smoking prevalence should focus on promoting smoking cessation, especially among women and heavy smokers. On the other hand, they should improve interventions to prevent relapse, targeting young people abstinent for a short time and subjects affected by mild respiratory diseases.

## Figures and Tables

**Table 1:** Characteristics of responders and non-responders to the follow-up among current smokers and ex-smokers at the baseline.

	Baseline	Smokers		Baseline	Ex-Smokers	
	Responders 965 (51.5%)	Non-Responders 909 (48.5%)	*p*-Value	Responders 735 (63%)	Non-Responders 431 (37%)	*p*-Value
Centre			**<0.001**			**<0.001**
Verona–ISIA	305 (66.3)	155 (33.7)		323 (77.8)	92 (22.2)	
Verona–ISAYA	412 (59.2)	284 (40.8)		276 (64.6)	151 (35.4)	
Sassari–ISAYA	248 (34.5)	470 (65.5)		136 (42.0)	188 (58.0)	
Sex			**<0.001**			0.058
Men	519 (48.1)	561 (51.9)		379 (60.5)	247 (39.5)	
Women	446 (56.2)	348 (43.8)		356 (65.9)	184 (34.1)	
Age class (years)			**<0.001**			**<0.001**
20–31.2	313 (47.9)	340 (52.1)		122 (49.4)	125 (50.6)	
31.3–42.5	374 (50.1)	373 (49.9)		390 (63.6)	223 (36.4)	
42.6–53.9	278 (58.6)	196 (41.4)		222 (72.8)	83 (27.2)	
Occupation			**<0.001**			**<0.001**
Businessman	142 (50.5)	139 (49.5)		116 (60.4)	76 (39.6)	
Clerk	324 (56.2)	253 (43.8)		255 (65.1)	137 (34.9)	
Workman	187 (52.4)	170 (47.6)		141 (71.6)	56 (28.4)	
Unemployed	48 (33.6)	95 (66.4)		17 (34)	33 (66)	
Housewife	88 (55.3)	71 (44.7)		90 (67.7)	43 (32.3)	
Student	82 (48.0)	89 (52.0)		21 (42.9)	28 (57.1)	
Others	89 (50.3)	88 (49.7)		93 (62.4)	56 (37.6)	
Nasal allergies			0.625			0.884
No	790 (51.8)	734 (48.2)		563 (62.7)	335 (37.3)	
Yes	163 (50.3)	161 (49.7)		162 (63.3)	94 (36.7)	
Asthma			0.092			0.236
No	923 (51.8)	858 (48.2)		668 (62.3)	405 (37.7)	
Yes	35 (42.2)	48 (57.8)		57 (69.5)	25 (30.5)	
Chronic bronchitis			**0.033**			0.182
No	772 (52.8)	690 (47.2)		634 (63.5)	364 (36.5)	
Yes	178 (46.6)	204 (53.4)		82 (57.7)	60 (42.3)	
Cigarette smoked daily	10 (7–20)	15 (10–20)	**<0.001**	10 (6–20)	10 (6–20)	0.557
Age at smoking initiation	17 (15–19)	17 (15–18)	0.380	----	----	----
Pack-years	9.0 (4.1–18.1)	9.4 (4.5–17.0)	0.737	12.6 (6.3–21)	15.1 (5.1–27.7)	0.872
Abstinence years	-----	-----	-----	8.4 (3.1–13.9)	6.5 (2.3–11.3)	**0.002**

Non-responders comprised people who either did not return the whole questionnaire or did not answer the question on smoking habits (item non-responders). Categorical variables are presented as absolute values with percent values in parentheses, while continuous variables are presented as median (25th percentile–75th percentile). *p*-values were computed by Fisher’s exact test or chi-squared test for nominal variables, chi-square for trend for age, and Wilcoxon–Mann–Whitney for continuous variables. Significant results are highlighted in bold.

**Table 2:** Smoking cessation in a sample of 965 subjects who were current smokers at the baseline.

	Current Smokers at Baseline	Smoking Quitters (per cent)	*p*-Value
Centre			**0.008**
Verona-ISIA	305	78 (25.6)	
Verona-ISAYA	412	**149 (36.2)**	
Sassari-ISAYA	248	**85 (34.3)**	
Sex			0.129
Male	519	179 (34.5)	
Female	446	133 (29.8)	
Age class (years old)			**<0.001**
20–31.2	361	**144 (39.9)**	
31.3–42.5	412	118 (28.6)	
42.6–53.9	192	50 (26.0)	
Occupation			**0.009**
Businessman	142	46 (32.4)	
Clerk	324	107 (33.0)	
Workman	187	64 (34.2)	
Unemployed	48	15 (31.2)	
Housewife	88	22 (25)	
Student	82	**39 (47.6)**	
Retired and others	89	18 (20.2)	
Allergic rhinitis			0.097
No	790	245 (31.0)	
Yes	163	62 (38.0)	
Asthma			**0.043**
No	923	293 (31.7)	
Yes	35	**17 (48.6)**	
Chronic bronchitis			0.510
No	772	254 (32.9)	
Yes	178	54 (30.3)	
Cigarettes smoked daily			**<0.001**
1–5	195	**71 (36.4)**	
6–10	295	**114 (38.6)**	
11–15	182	48 (26.4)	
16–40	263	56 (21.3)	
Age of smoking initiation			0.860
8–15 years old	252	73 (29.0)	
16–17 years old	262	73 (27.9)	
18–19 years old	207	58 (28.0)	
>20 years old	185	58 (31.4)	
Pack-years			**<0.001**
<5	272	**99 (36.4)**	
(5–10)	211	**69 (32.7)**	
(10–15)	141	42 (29.8)	
(15–80)	274	50 (18.2)	

*p*-values were computed by Fisher’s exact test or chi-squared test for categorical variables and by chi-square for trend for continuous categorised variables (age, cigarettes smoked daily, age at smoking initiation, and pack-years). Significant *p*-values are highlighted in bold, as well as the corresponding highest percentages.

**Table 3:** Influence of socio-demographic characteristics, respiratory disorders, smoking intensity, and age at smoking initiation on smoking cessation.

	OR (95% CI)	*p*-Value
Centre		
Verona-ISIA	1	
**Verona-ISAYA**	**1.56 (1.42–1.72)**	**<0.001**
**Sassari-ISAYA**	**1.24 (1.13–1.36)**	**<0.001**
**Sex (women vs. men)**	**0.70 (0.54–0.91)**	**0.009**
Age class (years old)		
20–31.2	1	
**31.3–42.5**	**0.83 (0.72–0.95)**	**0.006**
40.6–53.9	0.89 (0.65–1.21)	0.444
**Asthma (Yes vs. No)**	**2.10 (1.03–4.31)**	**0.042**
Cigarettes smoked daily		
1–5	1	
6–10	1.24 (0.78–1.98)	0.352
11–15	0.70 (0.44–1.11)	0.130
**16–40**	**0.55 (0.33–0.92)**	**0.022**
Age of smoking initiation		
8–15	1	
16–17	0.87 (0.70–1.07)	0.176
18–19	0.83 (0.53–1.30)	0.408
>20	1.05 (0.88–1.25)	0.578

Odds Ratios (ORs) and *p-*values were computed by a multivariable logistic model. *p-*values were computed by the Wald test. Significant results are highlighted in bold.

**Table 4:** Smoking re-initiation in a sample of 735 ex-smokers at baseline.

	Ex-Smokers at Baseline	People Who Resumed Smoking (per cent)	*p*-Value
Centre			0.087
Verona-ISIA	323	31 (9.6)	
Verona-ISAYA	276	32 (11.6)	
Sassari-ISAYA	136	23 (16.9)	
Sex			0.819
Male	379	43 (11.3)	
Female	356	43 (12.1)	
Age class (years old)			**<0.001**
**20–31.** **2**	122	**22 (18)**	
31.3–42.5	390	52 (13.3)	
**42.6**–53.9	222	12 (5.4)	
Occupation			0.222
Businessman	116	17 (14.7)	
Clerk	255	28 (11)	
Workman	141	17 (12.1)	
Unemployed	17	5 (29.4)	
Housewife	90	9 (10)	
Student	21	3 (14.3)	
Retired and others	93	7 (7.53)	
**Allergic rhinitis**			**0.038**
No	563	59 (10.5)	
**Yes**	162	**27 (16.7)**	
Asthma			0.528
No	688	78 (11.7)	
Yes	57	8 (14)	
**Chronic bronchitis**			**0.006**
No	634	66 (10.4)	
**Yes**	82	**17 (20.7)**	
Cigarettes smoked daily			0.902
1–5	148	11 (7.43)	
6–10	216	39 (18.1)	
11–15	117	10 (8.5)	
16–40	187	23 (12.3)	
Age of smoking initiation			0.477
1–15 years old	225	28 (12.4)	
16–17 years old	195	19 (9.7)	
18–19 years old	156	23 (14.7)	
>20 years old	105	15 (14.3)	
Pack-years			0.087
<5	170	27 (15.9)	
5–9	87	25 (28.7)	
10–14	58	15 (25.9)	
15–80	56	12 (21.4)	
**Abstinence duration (years)**			**<0.001**
<3 years	153	**42 (27.5)**	
3–7.4 years	140	20 (14.3)	
7.5–13.4 years	167	13 (7.8)	
>13.5 years	169	6 (3.6)	

*p*-values were computed by Fisher’s exact test or chi-squared test for categorical variables, and by chi-square for trend for continuous categorised variables (age, cigarettes smoked daily, age at smoking initiation, and pack-years). Significant results are highlighted in bold.

**Table 5:** The influence of socio-demographic characteristics, respiratory disorders, baseline smoking intensity, and abstinence duration on smoking re-initiation.

	OR (95% CI)	*p*-Value
Centre		
Verona-ISIA	1	
**Verona-ISAYA**	**0.86 (0.80–0.91)**	**<0.001**
Sassari-ISAYA	1.08 (0.97–1.20)	0.170
Sex (women vs. men)	1.18 (0.77–1.79)	0.448
Age class (years old)		
20–31.2	1	
31.3–42.5	0.99 (0.57–1.71)	0.961
42.6–53.9	**0.34 (0.22–0.54)**	**<0.001**
Asthma (Yes vs. No)	1.22 (0.63–2.37)	0.559
Cigarettes smoked daily		
1–5	1	
**6–10**	**3.19 (2.01–5.07)**	**<0.001**
11–15	1.26 (0.58–2.73)	0.550
**16–40**	**2.41 (1.41–4.14)**	**<0.001**
Abstinence duration (years)		
<3 years	1	
**3–7.4 years**	**0.46 (0.34–0.63)**	**<0.001**
**7.5–13.4 years**	**0.23 (0.18–0.29)**	**<0.001**
**>13.5 years**	**0.15 (0.06–0.36)**	**<0.001**

## Data Availability

Not applicable.

## Availability of Data and Materials

The datasets analysed during the current study will be available as Additional files upon manuscript acceptance.

## Abbreviations

COPD, Chronic obstructive pulmonary disease; ECRHS, European Community Respiratory Health Survey; GEIRD, Gene-Environment Interactions in Respiratory Diseases; ISAYA, Italian Study on Asthma in Young Adults; ISIA, Italian Study on the Incidence of Asthma; ITC, International Tobacco Control; NESARC, National Epidemiologic Survey of Alcohol and Related Conditions; PASSI, Italian behavioral risk factor surveillance system; WHO, World Health Organization.

## References

[1] (2017). WHO Report on the Global Tobacco Epidemic, 2017: Monitoring Tobacco Use and Prevention Policies.

[2] World Health Organization (2018). Who Global Report on Trends in Prevalence of Tobacco Smoking 2000–2025.

[3] Sun R., Mendez D. (2019). Finding the optimal mix of smoking initiation and cessation interventions to reduce smoking prevalence. PLoS ONE.

[4] Castro Y., Cano M.A., Businelle M.S., Correa-Fernandez V., Wetter D.W. (2014). A cross-lagged analysis of five intrapersonal determinants of smoking abstinence. Drug Alcohol Depend..

[5] Kong G., Camenga D., Krishnan-Sarin S. (2012). Parental influence on adolescent smoking cessation: Is there a gender difference?. Addict. Behav..

[6] Jackson S.E., Steptoe A., Wardle J. (2015). The influence of Partner’s behavior on health behavior change: The English longitudinal study of Ageing. JAMA Intern. Med..

[7] Filippidis F.T., Gerovasili V., Vardavas C.I., Agaku I.T., Tountas Y. (2014). Determinants of use of smoking cessation aids in 27 European countries. Prev. Med..

[8] Gallus S., Muttarak R., Franchi M., Pacifici R., Colombo P., Boffetta P., Leon M.E., La Vecchia C. (2013). Why do smokers quit?. Eur. J. Cancer Prev..

[9] Smedberg J., Lupattelli A., Mårdby A.C., Nordeng H. (2014). Characteristics of women who continue smoking during pregnancy: A cross-sectional study of pregnant women and new mothers in 15 European countries. BMC Pregnancy Childbirth.

[10] Stegberg M., Hasselgren M., Montgomery S., Lisspers K., Ställberg B., Janson C., Sundh J. (2018). Changes in smoking prevalence and cessation support, and factors associated with successful smoking cessation in Swedish patients with asthma and COPD. Eur. Clin. Respir. J..

[11] Holm M., Schiöler L., Andersson E., Forsberg B., Gislason T., Janson C., Jogi R., Schlünssen V., Svanes C., Torén K. (2017). Predictors of smoking cessation: A longitudinal study in a large cohort of smokers. Respir. Med..

[12] Bush T., Zbikowski S.M., Mahoney L., Deprey M., Mowery P., Cerutti B. (2012). State quitlines and cessation patterns among adults with selected chronic diseases in 15 states, 2005–2008. Prev. Chronic Dis..

[13] Hylkema M.N., Sterk P.J., De Boer W.I., Postma D.S. (2007). Tobacco use in relation to COPD and asthma. Eur. Respir. J..

[14] Jankowski M., Lawson J.A., Shpakou A., Poznański M., Zielonka T.M., Klimatckaia L., Loginovich Y., Rachel M., Gereová J., Minarowski Ł. (2020). Smoking cessation and vaping cessation attempts among cigarette smokers and E-cigarette users in Central and Eastern Europe. Int. J. Environ. Res. Public Health.

[15] Hilberink S.R., Jacobs J.E., Schlösser M., Grol R.P.T.M., De Vries H. (2006). Characteristics of patients with COPD in three motivational stages related to smoking cessation. Patient Educ. Couns..

[16] Melzer A.C., Feemster L.C., Crothers K., Carson S.S., Gillespie S.E., Henderson A.G., Krishnan J.A., Lindenauer P.K., McBurnie M.A., Mularski R.A. (2016). Respiratory and bronchitic symptoms predict intention to quit smoking among current smokers with, and at risk for, chronic obstructive pulmonary disease. Ann. Am. Thorac. Soc..

[17] Masaoka H., Gallus S., Ito H., Watanabe M., Yokomizo A., Eto M., Matsuo K. (2017). Aldehyde dehydrogenase 2 polymorphism is a predictor of smoking cessation. Nicotine Tob. Res..

[18] Jeong Yang J., Song M., Yoon H.S., Lee H.W., Lee Y., Lee S.A., Choi J.Y., Lee J.K., Kang D. (2015). What are the major determinants in the success of smoking cessation: Results from the health examinees study. PLoS ONE.

[19] García-Rodríguez O., Secades-Villa R., Flórez-Salamanca L., Okuda M., Liu S.M., Blanco C. (2013). Probability and predictors of relapse to smoking: Results of the National Epidemiologic Survey on Alcohol and Related Conditions (NESARC). Drug Alcohol Depend..

[20] Wetter D.W., Cofta-Gunn L., Fouladi R.T., Cinciripini P.M., Sui D., Gritz E.R. (2004). Late relapse/sustained abstinence among former smokers: A longitudinal study. Prev. Med..

[21] Herd N., Borland R., Hyland A. (2009). Predictors of smoking relapse by duration of abstinence: Findings from the International Tobacco control (ITC) four country survey. Addiction.

[22] Gökbayrak N.S., Paiva A.L., Blissmer B.J., Prochaska J.O. (2015). Predictors of relapse among smokers: Transtheoretical effort variables, demographics, and smoking severity. Addict. Behav..

[23] Caraballo R.S., Kruger J., Asman K., Pederson L., Widome R., Kiefe C.I., Hitsman B., Jacobs D.R. (2014). Relapse among Cigarette Smokers: The CARDIA longitudinal study—1985–2011. Addict. Behav..

[24] Kukkamalla M.A., Pentapati K.C., Suresh G., Goyal R., Cornelio S.M. (2013). Smoking re-initiation after cessation program: Comparison of associated factors between young and older adults. J. Nat. Sci. Biol. Med..

[25] Piñeiro B., López-Durán A., Martínez-Vispo C., Fernández del Río E., Martínez Ú., Rodríguez-Cano R., Míguez M.C., Becoña E. (2017). Smoking relapse situations among a community-recruited sample of Spanish daily smokers. Addict. Behav..

[26] Blok D.J., De Vlas S.J., Van Empelen P., Van Lenthe F.J. (2017). The role of smoking in social networks on smoking cessation and relapse among adults: A longitudinal study. Prev. Med..

[27] Moore S., Teixeira A., Stewart S. (2014). Effect of network social capital on the chances of smoking relapse: A two-year follow-up study of urban-dwelling adults. Am. J. Public Health.

[28] Hajek P., Stead L.F., West R., Jarvis M., Hartmann-Boyce J., Lancaster T. (2013). Relapse prevention interventions for smoking cessation. Cochrane Database Syst. Rev..

[29] Caponnetto P., Polosa R. (2017). Are we addressing relevant determinants of smoking cessation?. Eur. Respir. J..

[30] Verlato G., Nguyen G., Marchetti P., Accordini S., Marcon A., Marconcini R., Bono R., Fois A., Pirina P., De Marco R. (2016). Smoking and new-onset asthma in a prospective study on Italian adults. Int. Arch. Allergy Immunol..

[31] Verlato G., Melotti R., Corsico A.G., Bugiani M., Carrozzi L., Marinoni A., Dallari R., Pirina P., Struzzo P., Olivieri M. (2006). Time trends in smoking habits among Italian young adults. Respir. Med..

[32] Verlato G., Corsico A., Villani S., Cerveri I., Migliore E., Accordini S., Carolei A., Piccioni P., Bugiani M., Cascio V.L. (2003). Is the prevalence of adult asthma and allergic rhinitis still increasing? Results of an Italian study. J. Allergy Clin. Immunol..

[33] De Marco R., Zanolin M.E., Accordini S., Signorelli D., Marinoni A., Bugiani M., Cascio V., Woods R., Burn P. (1999). A new questionnaire for the repeat of the first stage of the European Community Respiratory Health Survey: A pilot study. Eur. Respir. J..

[34] De Marco R., Accordini S., Antonicelli L., Bellia V., Bettin M.D., Bombieri C., Bonifazi F., Bugiani M., Carosso A., Casali L. (2010). The gene-environment interactions in respiratory diseases (GEIRD) project for the GEIRD study group. Int. Arch. Allergy Immunol..

[35] Olivieri M., Poli A., Marco RDe Zuccaro P., Ferrari M., Lampronti G., De Marco R., Cascio V.L., Pacifici R. (2002). Tobacco Smoke Exposure and Serum Cotinine in a Random Sample of Adults Living in Verona, Italy. Arch. Environ. Health.

[36] Compalati E., Ridolo E., Passalacqua G., Braido F., Villa E., Canonica G.W. (2010). The link between allergic rhinitis and asthma: The united airways disease. Expert Rev. Clin. Immunol..

[37] Hosmer D.W., Lemeshow S., Sturdivant R.X. (2013). Applied Logistic Regression.

[38] Coppo A., Baldissera S., Migliardi A., Minardi V., Quarchioni E., Ferrante G., Dal Molin A., Faggiano F., PASSI Working Group (2017). Quit attempts and smoking cessation in Italian adults (25–64 years): Factors associated with attempts and successes. Eur. J. Public Health.

[39] Samim D., Méan M., Clair C., Marques-Vidal P. (2018). A 10-year observational study on the trends and determinants of smoking status. PLoS ONE.

[40] Pesce G., Marcon A., Calciano L., Perret J.L., Abramson M.J., Bono R., Bousquet J., Fois A.G., Janson C., Jarvis D. (2019). Time and age trends in smoking cessation in Europe. PLoS ONE.

[41] Verlato G., Melotti R., Olivieri M., Corsico A., Bugiani M., Accordini S., Villani S., Migliore E., Marinoni A., Pirina P. (2010). Asthmatics and ex-smokers respond early, heavy smokers respond late to mailed surveys in Italy. Respir. Med..

[42] Johannessen A., Verlato G., Benediktsdottir B., Forsberg B., Franklin K., Gislason T., Villani S., Migliore E., Marinoni A., Pirina P. (2014). Longterm follow-up in European respiratory health studies—patterns and implications. BMC Pulm. Med..

[43] Everard C.D., Silveira M.L., Kimmel H.L., Marshall D., Blanco C., Compton W.M. (2020). Association of Electronic Nicotine Delivery System Use With Cigarette Smoking Relapse Among Former Smokers in the United States. JAMA Netw. Open.

